# Impact of [^64^Cu][Cu(ATSM)] PET/CT in the evaluation of hypoxia in a patient with Glioblastoma: a case report

**DOI:** 10.1186/s12885-019-6368-8

**Published:** 2019-12-06

**Authors:** Vincenzo Gangemi, Chiara Mignogna, Giusy Guzzi, Angelo Lavano, Salvatore Bongarzone, Giuseppe Lucio Cascini, Umberto Sabatini

**Affiliations:** 1 Department of Diagnostic Imaging, Nuclear Medicine Unit, Magna Graecia University of Catanzaro, Catanzaro, Italy; 2 Health Science - Interdipartimental Service Center, University “Magna Graecia” of Catanzaro Medical School, Catanzaro, Italy; 3 Department of Neurosurgery, Magna Graecia University of Catanzaro, Catanzaro, Italy; 4 School of Biomedical Engineering & Imaging Sciences, King’s College London, King’s Health Partners, St Thomas’ Hospital, London, SE1 7EH UK; 5 Neuroradiology Unit, University “Magna Graecia”, Catanzaro, Italy

## Abstract

**Background:**

Glioblastoma multiform (GBM), a malignant brain tumour, has a very often poor prognosis. The therapeutic approach is represented by surgery followed by radiotherapy and chemotherapy. Hypoxia is a factor that causes a reduction of both radiotherapy and chemotherapy effectiveness in GBM and other cancers. Through the use of [^64^Cu][Cu(ATSM)], a hypoxia-targeting positron emission tomography (PET) radiotracer, is possible to identify the presence of hypoxic areas within a lesion and therefore modulate the therapeutic approach according to the findings.

**Case presentation:**

In this case report, we observed an increase of radiotracer uptake from early acquisition to late acquisition in hypoxia sites and high correlation between [^64^Cu][Cu(ATSM) PET/CT results and expression of the hypoxia marker HIF-1α.

**Conclusions:**

[^64^Cu][Cu(ATSM) PET/CT represents a valid opportunity to reveal in vivo hypoxic areas in GBM lesion which can guide clinicians on selecting GMB patient’s therapeutic scheme.

## Background

Glioblastoma multiform (GBM) cases represent about 16% of all primary brain tumours in adults and 54% of the total anaplastic gliomas [1]. The actual standard therapeutic plan for GBM includes radical surgical removal combined with radio- and chemo-therapy. The median survival time for GBM patients is 3–4 months when only surgically treated. The adjuvant conventional radiotherapy prolongs three times the average survival time, with a three-year survival for only about 6% of patients. Radical radiotherapy alone or combined with chemotherapy (e.g. using Temozolomide) is recommended for patients not surgically treated or with residual cancer after surgery. Better survival has been reported in patients treated with combination of radio-chemotherapy compared with those receiving radiotherapy alone, indeed the total survival time increased from 12.1 to 14.6 months and the survival rate at 2 years increased from 10 to 26% [2, 3].

However, a major obstacle to GMB therapy is the presence of hypoxia that triggers cancer cell spreading into the healthy brain tissue which is the main cause of death in GBM patients [4].

Positron emission tomography/computed tomography (PET/CT) using [^64^Cu][Cu(ATSM)] has been used to identify hypoxic regions in GBM patients, giving the chance to plan more targeted treatments [5–7]. Interestingly, the tumour uptake of copper-62 radiolabelled ATSM ([^62^Cu][Cu(ATSM)]) in GMB patients is highly correlated with hypoxia-inducible factor 1α (HIF-1α) expression, a marker of tissue hypoxia.

Here, we present a case of a GBM patient who underwent a [^64^Cu][Cu(ATSM)] PET/CT study before surgery, then the pathological tissue following surgery was subjected to histological and HIF-1α immunohistochemical staining.

### Case presentation

**Fig. 1:**
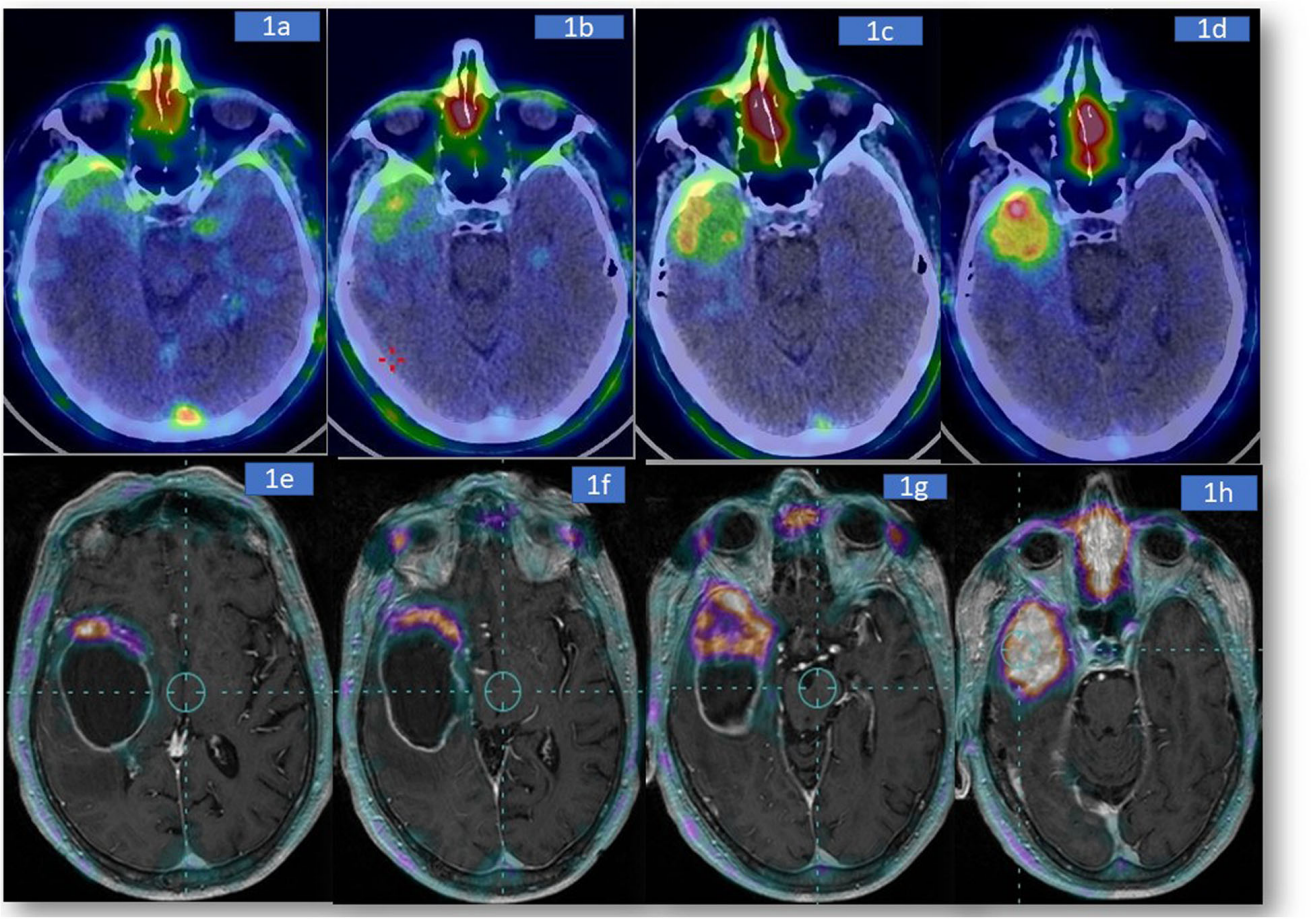
PET/CT acquisition at different times after injection. Brain images taken at 5 min (**a**) 1, (**b**), 4 (**c**) and 18 h (**d**) post-radiotracer injection. Progressive and significant uptake of [^64^Cu][Cu(ATSM)] into the lesion is documented during time. The SUVmax increases from 2.0 (5 min post-injection) to 4.9 (18 h post-injection). The heterogeneity of tumour is particular evident on PET-MRI fusion images obtained at 18 h. Images **e**-**h** represent different transaxial planes in cranio-caudal direction (upper (**e**) to lower (**h**) planes)

A 70-years-old Caucasian male, without relevant family or personal risk factors for neoplastic disease, suffered from severe headaches and nausea followed by a sudden episode of left leg weakness. On June 30th of 2016, magnetic resonance imaging (MRI) of the brain demonstrated a large heterogeneously enhancing tumour, with a diameter of 55 mm, localized in the right temporal lobe. The mass showed non-enhancing central fluid signal component suggesting central necrosis. There was surrounding edema with mass effect. On July 07th of 2016, the patient was in a confused state, responsive to verbal stimulation, anterograde amnesia, hypoplastic facies and depression. On July 12th of 2016, the examination [^64^Cu][Cu(ATSM)] PET/CT brain images showed “pathological accumulation of the radiopharmaceutical at the level of right temporopolar brain region; in particular the qualitative increment of tracer uptake from early to late scan was evident and sustained by a progressive increase of SUVmax with time, reaching a peak SUVmax value at approximately 18 h after initial [^64^Cu][Cu(ATSM)] administration (SUVmax of 3.2, 4.1 and 4.9 at 1, 4 and 18 h, respectively, Fig. 1a-d).

We found that [^64^Cu][Cu(ATSM)] uptake is 1.8 times more intense in the lower part of tumour (temporal section) than the upper part; SUVmax at 18 h post-radiotracer injection in the lower and upper parts were 4.9 (Fig. 1h) and 2.7 (Fig. 1g), respectively.

**Fig. 2:**
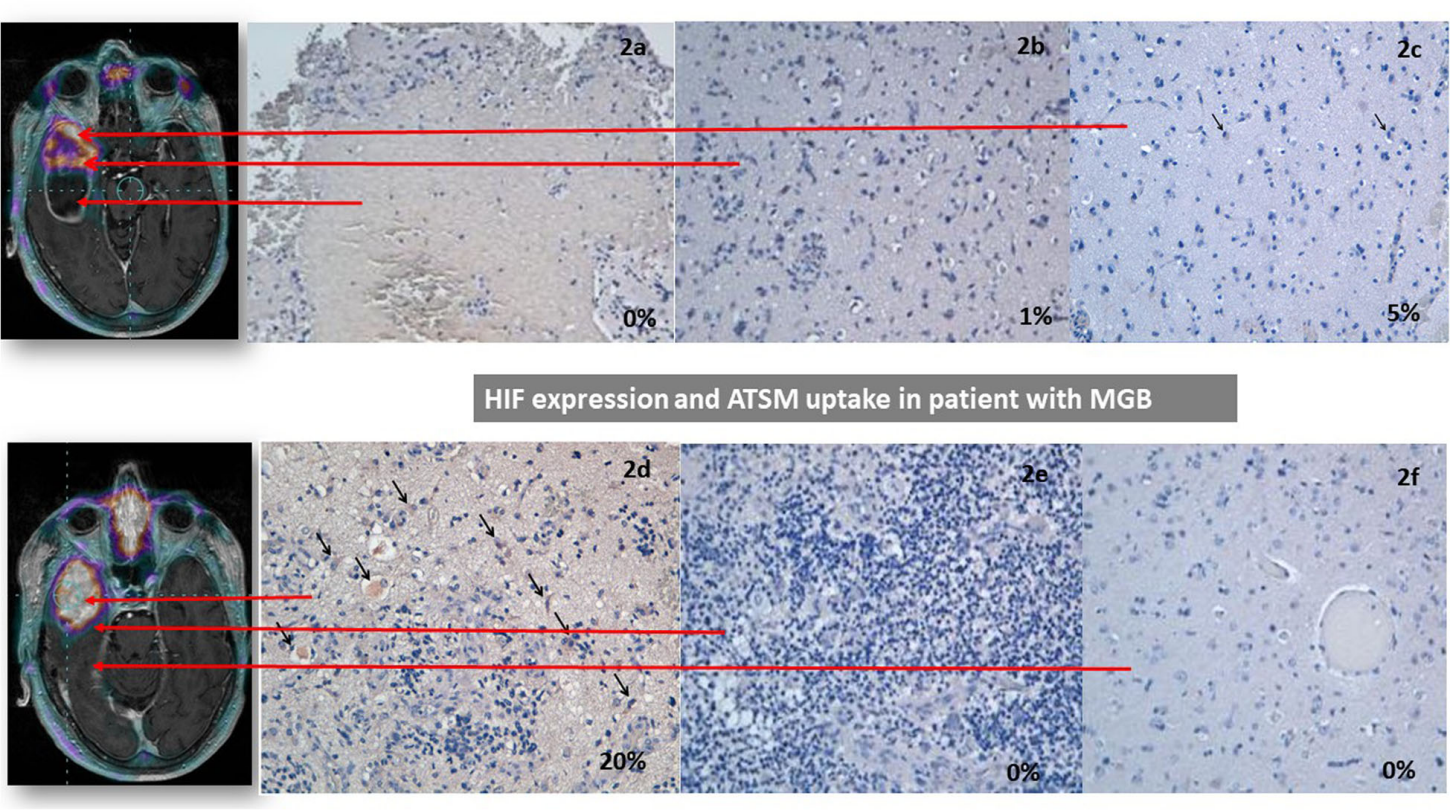
Spatial correlation between [^64^Cu][Cu(ATSM)] PET images and HIF-1α expression. **a** Necrotic area surrounded by neoplastic cells, no HIF-1α signal was detected. **b** High cellular neoplastic area, 1% of positive cells to HIF-1α. **c** Peripheral tumour area with lower cellularity, 5% of positive cells to HIF-1α corresponding to SUVmax of 2.7. **d** Central neoplastic area with HIF-1α expression in 20% of the cells (arrows) corresponding to SUVmax of 4.9. **e** Neoplastic area associated inflammatory infiltrate, no HIF-1α positive cells observed. **f**. Peripheral area adjacent to the tumour, reactive gliosis. Absent expression of HIF-1α. **a**-**f** The selection of the regions is based on manual reporting from the neurosurgeons

On July 15th of 2016, the patient underwent surgical removal of the right brain temporal lesion, then tissues were subjected to histological and immunohistochemical analysis for HIF-1α expression. We found that region with high [^64^Cu][Cu(ATSM)] uptake showed the highest immunohistologic expression of HIF-1α (Fig. 2d).

On July 20th and 22nd of 2016, a postoperative brain CT scan demonstrated the radical resection cavity, characterized by hypodensity (due to structural tissue changes determined by local manipulation), decrease of the previous mass effect and mid-line shift. On July 23rd of 2016, the patient returned home with a residual spatial and temporal disorientation.

## Methods

Brain PET/CT study was performed at 5 min, 1, 4 and 18 h after intravenous injection of 314 MBq [^64^Cu][Cu(ATSM)] (Acom srl, Macerata Italy). Ten minutes scans were acquired by using a PET/CT 2D (General Electrics, Discovery ST8).

The presence of intense radiotracer uptake corresponding to tumour lesion, higher than background was considered positive for tumour hypoxia. SUVmax was measured by using a circular region of interest positioned on the part of tumour with higher uptake; this analysis was repeated for all PET/CT scans.

The removed lesion was particularly wide, and slices of tumour were obtained to produce axial sections in cranio-caudal direction based on MRI brain scan. Clearly tissue planes were deformed respect to MRI sections. Neurosurgeons have selected three samples for one representative axial plane: in the central-inner part, at the tumour margin, and in the normal surrounding tissue closely to the capsule. The sites of samples were manually reported on MRI-PET images by the neurosurgeons. Next, HIF-1α expression was performed. Although, this process might have produced a lack of spatial linearity between in vivo and ex vivo images, the relation respect to the tumour margin has been warranted.

Immunohistochemical staining procedures were carried out on formalin-fixed, paraffin-embedded cell blocks, representative of specific surgical regions, identified with surgical landmarks. For HIF-1α identification, a three-layer biotin-avidin-peroxidase system was utilized. Shortly, xylene dewaxed and alcohol-rehydrated serial tissue sections (4 μm-thick) were treated in EDTA buffer at 98 °C for 50 min, according to the antigen retrieval method. The standard streptavidin-biotin-peroxidase complex technique was performed, using sequential 30 min incubation with biotinylated linking antibody and peroxidase-labelled streptavidin (DAKO LSAB kit HRP, Carpinteria, CA). As a substrate chromogen solution for the development of the peroxidase activity, the 3,3′-diaminobenzidine (DAB, Vector Laboratories, Burlingame, U.S.A.) was used. After a slight nuclear counterstaining performed with haematoxylin, sections were then cover-slipped with a synthetic mounting medium (Entellan, Merck, Germany).

Cells showing a definite brown staining were counted in 10 high-magnification random fields of the most representative areas of the tumours, the transition zone between tumour and peripheral (non-neoplastic) areas. Results were expressed as the percentage of positive cells among the total number of cells.

## Discussion and conclusions

GBM is a very aggressive tumour which has a poor prognosis, standard therapeutic strategy includes a radical surgical removal of glioma combined with radio- and chemo-therapy. However, the effectiveness of radiotherapy and chemotherapy is compromised by the presence of hypoxia [8].

The factors playing a major role in hypoxia development are the increased demand for oxygen from cancer cells in growth and a reduction of the blood oxygen transport derived from spatial disorganization of tumour vascular networks [6]. Hypoxia decreases patients survival and induces resistance in GBM cancer cells to radiotherapy and chemotherapy treatments [6–11].

[^64^Cu][Cu(ATSM)] PET/CT is a minimal invasive molecular imaging technique able to accurately map the areas of hypoxia [12, 13]. Previous PET studies showed that high uptake of [^64^Cu][Cu(ATSM)] correlates with the treatment response and poor diagnosis [8]. The identification of hypoxic areas is a main goal for an accurate planning treatment in GBM patients. [^64^Cu][Cu(ATSM)] is a lipophilic molecule with high cell membrane permeability and diffuses readily through the bloodstream within the cell; here it is reduced from copper (II) to copper (I) and then trapped in the hypoxic tissues but not in the normal tissues [13]. In agreement with preclinical and clinical studies [10, 14], our case represents an in vivo demonstration of [^64^Cu][Cu(ATSM)] selectivity for hypoxic tumour tissue, confirmed by immunohistochemical analysis, proving itself as a successful diagnostic modality for the detection of GBM hypoxia. The detection of hypoxia using [^64^Cu][Cu(ATSM)] could lead to a more efficient tumour planning therapy in GBM patients.

## Data Availability

Not applicable.

## Abbreviations

[^64^Cu][Cu(ATSM)]: Copper-64-diacetyl-bis(N^4^-methylthiosemicarbazone)
GBM: Glioblastoma multiform
MRI: Magnetic resonance imaging
PET: Positron emission tomography
PET/CT: Positron emission tomography/computed tomography
